# Disentangling the Phylogenetic and Ecological Components of Spider Phenotypic Variation

**DOI:** 10.1371/journal.pone.0089314

**Published:** 2014-02-19

**Authors:** Thiago Gonçalves-Souza, José Alexandre Felizola Diniz-Filho, Gustavo Quevedo Romero

**Affiliations:** 1 Programa de Pós-Graduação em Biologia Animal, Departamento de Zoologia e Botânica, Universidade Estadual Paulista, São José do Rio Preto, SP, Brasil; 2 Departamento de Ecologia, Instituto de Ciências Biológicas, Universidade Federal de Goiás, Goiânia, GO, Brasil; 3 Departamento de Biologia Animal, Instituto de Biologia, Universidade Estadual de Campinas, Campinas, SP, Brasil; CNRS, Université de Bourgogne, France

## Abstract

An understanding of how the degree of phylogenetic relatedness influences the ecological similarity among species is crucial to inferring the mechanisms governing the assembly of communities. We evaluated the relative importance of spider phylogenetic relationships and ecological niche (plant morphological variables) to the variation in spider body size and shape by comparing spiders at different scales: (i) between bromeliads and dicot plants (i.e., habitat scale) and (ii) among bromeliads with distinct architectural features (i.e., microhabitat scale). We partitioned the interspecific variation in body size and shape into phylogenetic (that express trait values as expected by phylogenetic relationships among species) and ecological components (that express trait values independent of phylogenetic relationships). At the habitat scale, bromeliad spiders were larger and flatter than spiders associated with the surrounding dicots. At this scale, plant morphology sorted out close related spiders. Our results showed that spider flatness is phylogenetically clustered at the habitat scale, whereas it is phylogenetically overdispersed at the microhabitat scale, although phylogenic signal is present in both scales. Taken together, these results suggest that whereas at the habitat scale selective colonization affect spider body size and shape, at fine scales both selective colonization and adaptive evolution determine spider body shape. By partitioning the phylogenetic and ecological components of phenotypic variation, we were able to disentangle the evolutionary history of distinct spider traits and show that plant architecture plays a role in the evolution of spider body size and shape. We also discussed the relevance in considering multiple scales when studying phylogenetic community structure.

## Introduction

A non-random distribution of animal body sizes along resource gradients results of the interplay between environmental and behavioral traits. Life-history theory predicts that traits maximizing fitness in a particular selective environment are maintained along evolutionary history of an organism [1]. For instance, the morphological characteristics (e.g., smaller species) of species that are evolutionary conserved will favor the selective colonization of vegetation habitats habitat structure [2]. However, most studies considering the relationship between morphology and ecology fail to take phylogeny into account. Since closely related species tend to share similar morphology and ecological niches, not taking phylogeny into account explicitly treats them as independent observations [3], obscuring the variation among species due to common ancestry. The integration of phylogeny into community ecology provides a historical framework within which to understand the contributions of ecological and evolutionary processes in dictating the contemporary distributions of species [4].

Plant-living spiders are a good system for studying the relationship between ecological niche and morphology since they have a prolonged, intimate relationship with individual plants [5]. For instance, plant traits could determine which taxa of spiders could live on a given plant, based on the prior match between spider morphology and plant architecture. Thus, we could expect that spiders that occur in plants with similar traits share similar body sizes because plant morphology has favored the selection of certain body size throughout evolutionary time. Accordingly, if some morphological traits are phylogenetically conserved, plant morphology will sort out close related spiders. Bromeliads are a good example of such plants, because they have a rosette-like architecture and a tight arrangement of leaves that are highly distinctive from dicot plants in the Neotropics, and thus favor long-term association with animals [6]. In fact, it has been shown that animals associated with bromeliads have more feeding opportunities compared to dicot-living relatives, as they can access both aquatic and terrestrial food sources [7]. Bromeliads could thus sort lineages of large spiders with higher energy requirements by selective colonization of spiders. Conversely, the arrangement of bromeliad leaves could favor species that are able to forage in this tight space, such as those spiders with flatter bodies. As a result, at microhabitat scale body shape is affected by adaptive evolution. In fact, it has been suggested that the decrease in some morphological characteristics generally enhances species' performance (e.g. sexual display, foraging), as predicted by the maneuverability hypothesis [8]. This enhanced foraging performance related to morphological adaptation was previously reported in other restrictive habitats, such as caves, rock crevices and dense habitats [9], [10]. The trade-off between energy requirements and forage performance could thus affect the evolution of spider body size and shape in distinct ways.

In this study we evaluated how plant species with distinct architectural features select for spider traits (body size and flatness) and how much of the variation in those traits is explained by the spiders' phylogeny vs. differences in plant architectural features. We evaluated whether spider body size is related to habitat constraints by comparing two adjacent habitat types, bromeliads and both herbaceous and shrubby vegetation (hereafter ‘dicot’). We also evaluated the effect of microhabitat characteristics by comparing spiders among 14 bromeliad species with distinctive architectural features. We addressed the following questions. Are bromeliad-living spiders larger and flatter than dicot-living spiders? Is spider body size related to bromeliad architecture? Do phylogenetic relatedness and ecological niches explain the phenotypic variation in spider body size and flatness among habitats and microhabitats? We used a phylogenetic comparative method to decompose the variation in spider body size and flatness into phylogenetic, niche conservatism and ecological components based on habitat and microhabitat as constraints of phenotypic variation. We used bromeliads and dicots as two different habitat types and the specific architectural features of bromeliad species as microhabitats.

## Materials and Methods

### Study Site and Organisms

This work was carried out at the Estação Biológica Santa Lúcia (EBSL) (19°57′S, 40°31′W; 600–900 m a.s.l.), an area of 440 ha in Santa Teresa, state of Espírito Santo, south-eastern Brazil. The vegetation of the EBSL is characterized as Atlantic Rainforest. At the EBSL, the Bromeliaceae family dominates many strata of the understory. In general, bromeliads make up large agglomerates of multispecific patches that naturally occur between forests and rocky outcrops, on shallow and structurally poor ground (hereafter named “bromeliad patches”) [11]. Small patches vary from 0.005 to 0.14 ha and large ones from 0.43 to 0.93 ha. The forest vegetation is dominated by members of the family Myrtaceae, Lauraceae, Sapotaceae, and Melastomataceae [12]. We classified the spider species in two guilds: active hunting spiders and web-building spiders (Table S1 in File S1). The basic difference between these guilds is the ability to weave webs. Although web building probably influences on feeding characteristics, we referred to these two groups as guilds, instead of feeding guilds.

### Data Survey

We sampled spiders in bromeliads and in both herbaceous and shrubby vegetation in nine bromeliad patches ranging from 125 m to 1031 m in distance from each other. The survey comprised 24 permanent plots surveyed over ten sampling periods at monthly intervals between February 2006 and September 2007. The number of plots per patch and plot size were proportional to the area of each patch. Plot size for bromeliad and ground samples was 7×3 m (n = 6) for small patches and 20×3 m (n = 18) for large patches. In bromeliad patches with at least two plots (n = 5 patches), each plot was 21 m from its nearest neighbor. We sampled terrestrial and epiphytic bromeliads (up to 1.5 m in height) in all plot areas and manually collected spiders on all plant foliage (dead and living leaves), the interiors of the rosettes and between the leaf axils of 1110 bromeliads comprising 32 species. Bromeliads sampling was performed using non-destructive methods. We fixed spiders in 75% ethanol for later identification. To minimize bias in the analyses, we only used the spiders from the 14 bromeliad species that matched the minimum abundance criteria of eight plants and six spiders per bromeliad species [13].

Spider density on vegetation is typically lower than on bromeliads, thus requiring us to increase the sampling effort (plot size); we used plots of 20×20 m (n = 18) in large patches and 20×7 m (n = 6) in small patches. The plots were 1 m apart; although this distance might not distinguish between two vegetation communities, plots at a distance of more than 1 m could include fauna from outside the bromeliad patch. The number of plots per bromeliad patch varied from one to five depending on the size of the patch. For example, we made a single 7×3 m plot for the smallest bromeliad patch (0.005 ha), whereas we made five plots of 20×3 m for the largest patch (0.93 ha). To avoid temporal discrepancies in comparative analysis, the three habitat types were sampled concomitantly in each sampling period. We used beating trays to sample 20 herbaceous-shrubby plants from each large plot (n = 18) and 10 plants from each small plot (n = 6), which totaled 420 sampled plants. We sampled plants up to 3 m in height and the distance between them varied from 1 to 3 m. The beating trays were made up of a 1×1 m square wooden beam frame holding a 1 m^2^ cotton cloth; these trays were placed under the shrub and, with a stick, we beat the shrub 20 times so that the spiders would fall onto the cloth. Voucher specimens are deposited at the Instituto Butantan (Brazil). We obtained all necessary permits for the described field studies (provided by “Instituto Chico Mendes de Conservação da Biodiversidade/ICMBio-SISBIO”). We used different sampling methods for each habitat type to maximize spider collection.

### Spider Body Size Measurements

**Figure 1:**
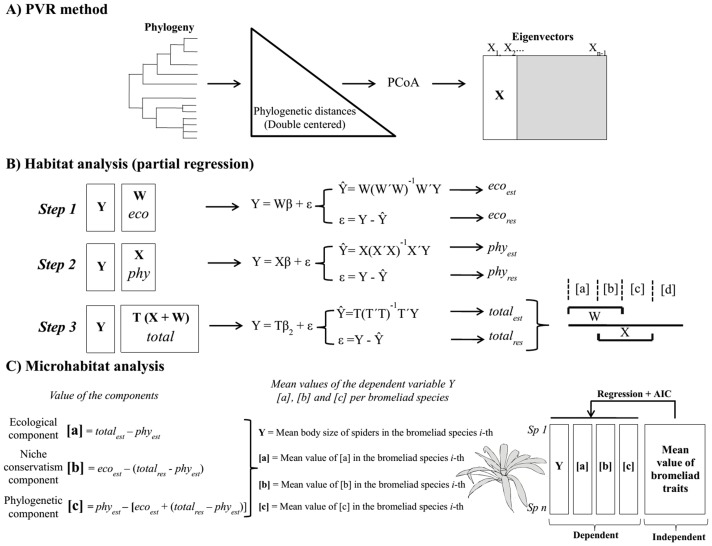
Consensus topology describing the phylogenetic relationships among spider species. Presence (black squares) and absence (white squares) of spider species in bromeliads (B) and/or dicot plants (D) (middle panel). The right panel shows the body size and flatness of species (grey bars) in logarithmic scale.

We took photographs and measured spider prosoma length and height as well as body length in a stereoscopic microscope (Leica MZ 16). We use prosoma length as a measure of body size and the ratio between prosoma height and body length as spider flatness (i.e. body shape). Because we were interested in understanding the associations of spider species with habitat types and bromeliad species, the body sizes of males and females were averaged for each spider species. We calculated the female/male prosoma size ratio from our data and did not find dimorphic species (range = 0.698–1.77, Table S2 in File S2). Thus, male-female body size pooling is unlikely to affect our conclusions. We built a supertree for spider species sampled based on topological relationships proposed in previous studies (see File S1 for detailed information on supertree building). Due to the lack of detailed information on branch lengths and difficulties in assigning dates to past lineage separations, which would be required for calibrating the phylogeny, we manually produced a consensus topology describing the phylogenetic relationships among species (Fig. 1). Because of this lack of resolution and definition regarding branch lengths, more refined inferences on evolutionary models are not adequate and we used a more statistical approach to analyze the relationships (see below) and interpret the results. We then used the programs PDTREE and PDDIST to draw the consensus phylogeny and calculate a pairwise patristic distance from which eigenvectors were extracted (see below) [14].

### Statistical Analyses

We partitioned the variance on the dependent variables (spider body size and flatness) using two groups of ecological predictors, guild (active hunting or web-building spiders), considered as an intrinsic ecological feature, and species habitat occupancy (bromeliad or dicot), as an extrinsic ecological feature. Before all analyses, the data on prosoma length and spider flatness were log transformed to meet test assumptions.

**Figure 2:**
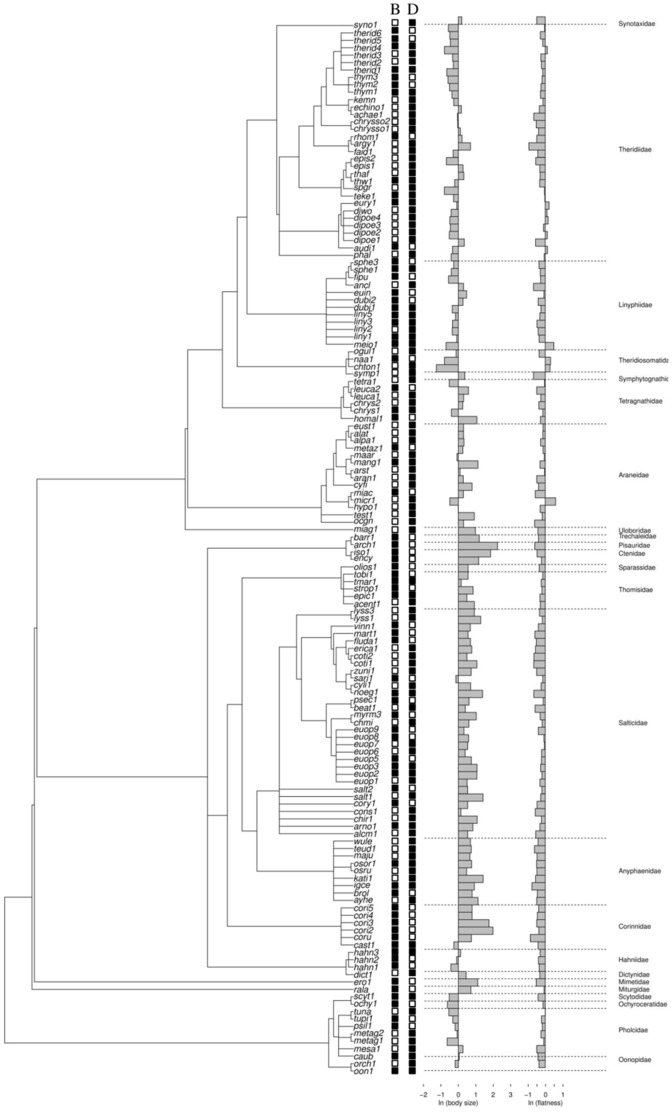
Schematic representation of the habitat and microhabitat analyses used to decompose the total variation in spider body size and flatness into phylogenetic, ecological and niche conservatism components. Phylogenetic eigenvector regression (PVR) is represented by a back-transformation of the phylogeny with a double-centralization of the resulting matrix and is followed by a principal coordinates analysis (PCoA); the matrix X represents the eigenvectors that are significantly correlated with species' body size (Fig. 2A). Figure 2B shows the partial regressions used to calculate components a, b, c and d; first, we calculated the estimated and residual values (*eco_est_* and *eco_res_*) for a regression between body size and the ecological data; then, we regressed body size and the phylogenetic data and saved the estimated and residual values (*phy_est_* and *phy_res_*); finally, we computed the regression between body size and both the ecological and the phylogenetic data to obtain the percentages of the variance explained (R^2^ of the regression method) by the ecological component [a], the niche conservatism b, the phylogenetic component [c] (phylogeny) and the unexplained variation d (unexplained variation), following the procedure proposed by Desdevises et al. [15]. Figure 2C illustrates the procedure used to obtain the mean value of spider body size (or flatness) and the average value of components [a], [b] and [c] obtained (see Figure 2B) for each bromeliad species. We then constructed a linear regression between each value (Y, [a], [b] and [c]) and the mean value of bromeliad morphological variables (leaf length, leaf width and number of leaves) after the selection of best models with the Akaike Information Criterion (AIC).

To account for the phylogenetic non-independence among species and test for phylogenetic effects, we used the approach proposed by Desdevises et al. [15], which is based on Phylogenetic Eigenvector Regression (PVR) analyses [16]. The original PVR method uses a Principal Coordinates Analysis (PCoA) to extract the eigenvectors of the phylogenetic distance matrix (D) after a double-centered transformation (Fig. 2A) [16]. The eigenvectors, which express phylogenetic differences among groups of species at distinct levels of the phylogeny, are then used as predictor variables in the partial regression (Fig. 2B) [15]. The R^2^ of these regressions express the amount of variation in trait explained by phylogenetic structure (or the effect of environment after partialling out the phylogenetic structure). Because PVR does not assume an explicit evolutionary model that would require a better knowledge of branch lengths (but see [17], [18]), it is an appropriate method for utilizing the unconfirmed and topology-based phylogenetic information available for our species.

In the original PVR procedure, the estimated (predicted) values of the multiple regression expresses the shared phylogenetic variance among species, thus representing an estimate of the phylogenetic component, whereas the residuals of the multiple regression express the proportion of the variation in Y (spider body size and flatness) that is independent of phylogeny (as expressed by the eigenvectors). The residuals of the model also called the specific component, can be viewed as the variation in Y independent of ancestral values, or at least less affected by the effects of phylogenetic variation at deeper scales, expressing variation of the trait Y closer to the tips of the phylogeny and resulting from recent adaptations [17], [19].

In the Desdevises et al. [15] generalization of PVR, both phylogenetic eigenvectors and ecological predictors are used to explain variation in a given trait Y. In the habitat analysis, we partitioned the variation in the response matrix Y (spider body size and flatness) between the ecological matrix (component a: habitat type or guild as dummy variables), the phylogenetic matrix obtained with the PVR analysis (component c), and the overlap of these effects (component b) following three steps: *step 1*, we regressed the response variable Y against the ecological variable (habitat type or guild – matrix W, Fig. 2B). The coefficient *R^2^_eco_* of this regression is equal to components a+b of the decomposition [15]. In this step, we retained the estimated (*eco_est_*) and residual (*eco_res_*) values for the microhabitat analysis (see below; Figs. 2B and 2C). *Step 2*, we regressed the response variable Y against the phylogenetic eigenvectors (matrix X, Fig. 2B) retained in a stepwise selection model following Desdevises et al. [15]. The coefficient *R^2^_phy_* of this regression is equal to components b+c of the decomposition. In this step, we retained the estimated (*phy_est_*) and residual (*phy_res_*) values for the microhabitat analysis (Figs. 2B and 2C). *Step 3*, we implemented a multiple regression on both ecological variables and phylogenetic eigenvectors. The coefficient *R^2^_tot_* represents the sum of components a, b, and c of the decomposition. At this step, we retained the estimated (*total_est_*) and residual (*total_res_*) values for the microhabitat analysis (Figs. 2B and 2C). After these three steps, we calculated the individual value of each component following the subtraction proposed by Desdevises et al. [15]: component a = *R^2^_tot_*−*R^2^_phy_*; component b = *R^2^_env_*+*R^2^_phy_*−*R^2^_tot_*; component c = *R^2^_tot_*−*R^2^_env_*. Component a is the ecological component, component b is the phylogenetically structured environmental variation, i.e., the “phylogenetic niche conservatism”, and component c is the phylogenetic component [15], [20]. We calculated the amount of unexplained (residual) variation d as 1−(a+b+c) [15].

A high and significant phylogenetic component c indicates that a given part of variation in Y is explained by phylogeny, independent of the ecological variables incorporated into the model. This can be interpreted as stochastic processes driving trait variation, the effects of past adaptations maintained by phylogenetic inertia of other non-measured plant morphological traits affecting spider body size and shape variation. If the variation in a morphological trait is related (*P*<0.05) to the ecological component in the partial regression, we consider that trait as phylogenetically overdispersed (i.e., species within the same habitat or guild are more distantly related). In contrast, if the variation in a morphological trait is significantly (*P*<0.05) related to the phylogenetic component, we considered that trait as phylogenetically clustered (i.e., species within the same habitat or guild are more closely related). As we used different morphological traits, it is possible that some traits have overdispersed distribution and others have clustered distribution. Thus, the decision between clustered and overdispersed pattern depends on the significance of the regression between each independent (ecology, phylogeny) and dependent (morphology) component and the morphological variable. We performed the partitioning method twice, once for habitat type (i.e. bromeliad or dicot) and once for guild.

At the microhabitat scale, we used the predicted and residual values obtained from the habitat analysis and regressed them against morphological variables of 14 bromeliad species (Fig. 2C). We performed the microhabitat analyses by using only spiders exclusively associated with bromeliads, and thus the ecological component was attributed only to guild. We used the retained predicted and residual values from the three regression analyses (steps 1–3, above) to extract the ecological, niche conservatism and phylogenetic components of the total variation. To obtain the ecological component [c] (related to guild information), we subtracted the values of “*total_res_*” from the values of “*phy_est_*” (Fig. 2C); to obtain the niche conservatism component, we subtracted the values of “*eco_est_*” from the values of “*total_res_*−*phy_est_*” (Fig. 2C). The niche conservatism component [b], i.e., the correlation of the ecological and phylogenetic components, represents the phylogenetically structured ecological variation [15]. To obtain the phylogenetic component [a], we subtracted the values of “*phy_est_*” from the values of “*eco_est_*+(*total_res_*−*phy_est_*)” (Fig. 2C). We used the phylogenetic (P), ecological (S) and niche conservatism (PS) components of phenotypic variation in the following analysis. As we collected spiders in 14 bromeliad species, we can calculate the mean value of spider body size and flatness by averaging spider morphological variables in the bromeliad species i-th. Similarly, the values of [a], [b] and [c] obtained from the predicted and residuals values of the partial regressions was averaged within each bromeliad species (Fig. 2C). Then, we considered the mean value of Y (body size or flatness), [a], [b] and [c] as dependent variables, and regressed them against the architectural variables of those bromeliads, i.e., the number of leaves, leaf width and length (Fig. 2C). However, we used only the variables retained in the model with the smallest Akaike Information Criterion value (Fig. 2C).

## Results

**Figure 3:**
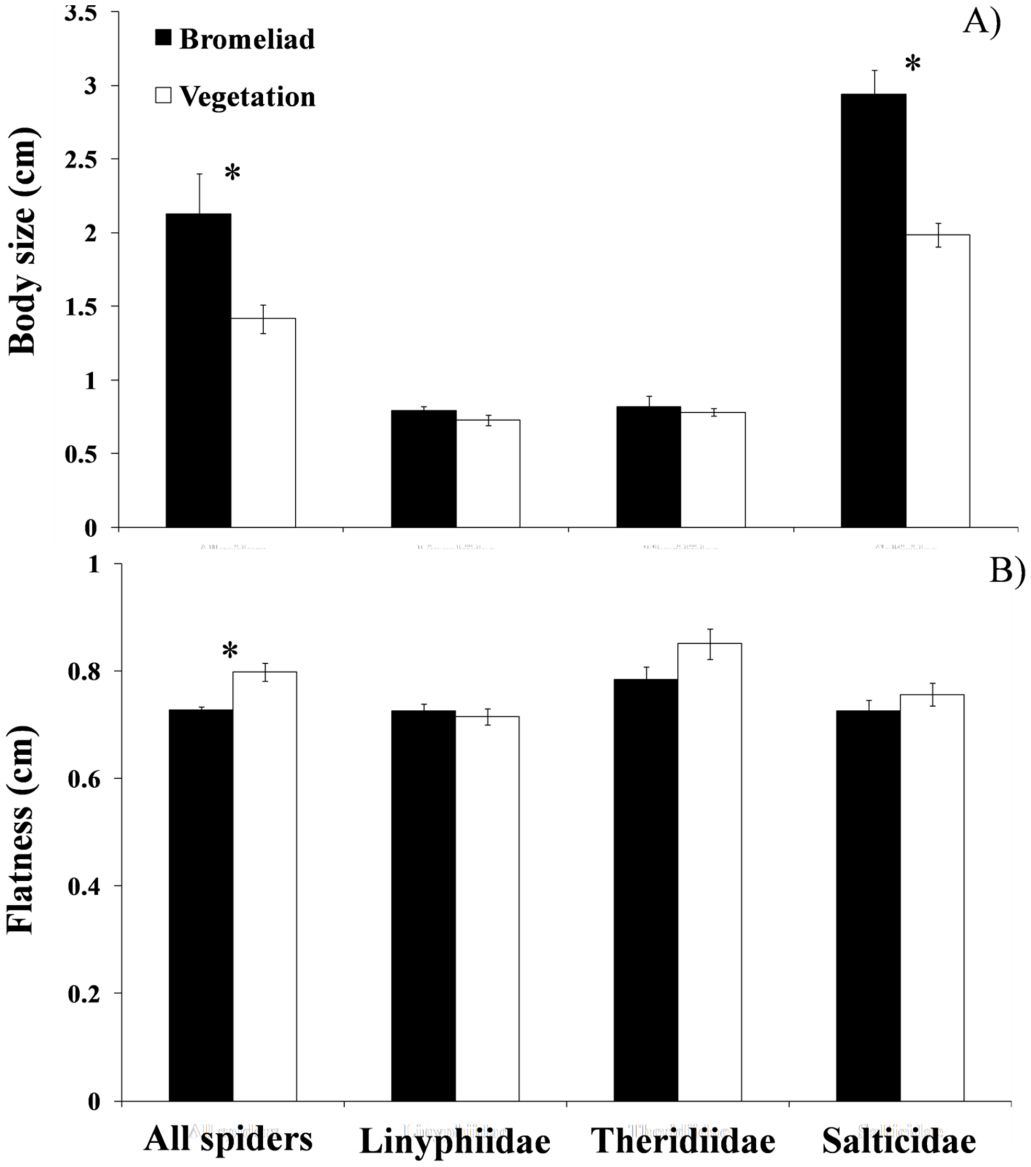
Average spider body size (A) and flatness (B) between bromeliads and surrounding dicots for all spiders, Linyphiidae, Theridiidae (both families of web-spiders) and Salticidae (hunting spiders). Error bars denote ± 1SE and asterisks indicate significant difference (*P*<0.05).

We found 145 spider species associated with bromeliad and dicot habitats. Of these, 117 species were exclusively associated with one plant type (47 were associated with bromeliads and 70 with dicots). Without controlling for phylogenetic relationships among species, we found that bromeliad-living spiders were on average 50% larger than dicot-living spiders (separate variances *t* test = 2.46; df = 58.81; *P* = 0.016; Fig. 3A). In addition, bromeliad spiders were 9% flatter than dicot spiders (separate variances *t* test = −4.03; df = 288,4; *P*<0.001; Fig. 3B).This effect is larger for hunting spiders (Salticidae) than for web-building ones; e.g., the bromeliad-living salticids were 47% larger than those salticids inhabiting dicots (separate variances *t* test = −5.24; df = 56.46; *P*<0.0001) (Fig. 3A). In contrast, the body size of web-building spiders, such as Linyphiidae (body size: separate variances *t* test = −1.267; df = 56.69; *P* = 0.21) and Theridiidae (separate variances *t* test = −0.489; df = 24.80; *P* = 0.628), was not affected by habitat type (Fig. 3A). The body flatness of these three families did not differ between bromeliads and dicots (Fig. 3B).

Partitioning out the total phenotypic variation of spiders at the habitat scale, we found phylogenetic signal in body size (guild, *R* ^2^ = 0.26; habitat, *R* ^2^ = 0.641) and flatness (guild, *R* ^2^ = 0.203; habitat, *R* ^2^ = 0.29), indicating that phenotypic similarity is related to spider phylogenetic relationships (Table 1). When comparing phenotypic variation among guilds we found that the niche conservatism component explained spider's body size (*R* ^2^ = 0.419) and flatness (*R* ^2^ = 0.106) (Table 1). Thus, this phylogenetically structured phenotypic variation suggests that body size and flatness are conserved in relation to species guild, an intrinsic ecological trait. However, when comparing phenotypic variation among habitats, the niche conservatism component weakly explained spider's body size (*R* ^2^ = 0.038) and flatness (*R* ^2^ = 0.02). The ecological component, by its turn, did not explain the variation in spider body size and flatness (Table 1).

**Table 1:** Coefficients of determination of partial regression models of spider morphological variables (body size and flatness) against phylogenetic (PVR eigenvectors) and ecological (habitat and guild) components.

Spider morphological characteristic	Phylogenetic component	Niche conservatism component	Ecological component	Unexplained variation
**Habitat**				
Body size	**0.641**	**0.038**	0.003	0.317
Flatness	**0.29**	0.02	0.001	0.69
**Guild**				
Body size	**0.26**	**0.419**	0.012	0.309
Flatness	**0.203**	**0.106**	0.002	0.689

These results represent the habitat scale analysis (Fig. 2B). Coefficients of determination in bold type denoting significant p-values (<0.05).

When we compared the variations in spider body size and flatness at the microhabitat scale without controlling for phylogeny, we found that the mean value of bromeliad-living spiders' body size was negatively correlated to leaf length (*R* ^2^_adj_ = 0.486, *P* = 0.015; Table 2) and the number of leaves (*P* = 0.045; Table 2). Partitioning out the variation in body size among bromeliad-living spiders, we found that the phylogenetic component is negatively related to leaf length and the number of leaves (*R* ^2^_adj_ = 0.785, *P*<0.001; Table 3), whereas the values of the ecological and niche conservatism components of spider body size were not related to microhabitat variables (Table 3). The absence of ecological and niche conservatism effects and the strong phylogenetic signal in spider body size suggest that spider phylogenetic relationships is affected by bromeliad morphological characteristics. It appears that bromeliads with elongated leaves selected for small-bodied spider clades. Conversely, the phylogenetic and ecological components of spider flatness were both related to bromeliad leaf length. The mean ecological component (component *a*) of spider flatness was negatively correlated with bromeliad leaf length (*R* ^2^_adj_ = 0.321, *P* = 0.037; Table 3), while the mean phylogenetic component was positively related to leaf length (*R* ^2^_adj_ = 0.427, *P* = 0.029; Table 3), which means that spiders that occur in elongated bromeliads were flatter. The mean niche conservation component was not related to any of the bromeliad variables (Table 3).

**Table 2:** Linear regression analysis of body size and flatness against bromeliad variables without considering phylogenetic information.

	Bromeliad variables	*β*	R^2^_adj_	*P*
**Body size**	LL	**−0.617**	**0.486**	**0.015**
	LW	-	-	-
	NL	**−0.481**	**0.486**	**0.045**
**Flatness**	LL	0.502	0.252	0.067
	LW	-	-	-
	NL	-	-	-

The regression analysis was made only with the bromeliad variables retained as the best model based on the Akaike Information Criterion. The R^2^_adj_ value presents the explained variation of the best model for each component regressed against bromeliad morphological variables. Thus, the repeated R^2^_adj_ value does not represent the explained variation of each variable.

**Table 3:** Linear regressions of body size and flatness and their partitioned components from phylogenetic eigenvector regression analysis (PVR) against leaf length (LL), leaf width (LW), and number of leaves (NL).

	Bromeliad variables	EC			NCC			PC		
		*β*	R^2^_adj_	*P*	*β*	R^2^_adj_	*P*	*β*	R^2^_adj_	*P*
**Body size**	LL	-	-	-	-	-	-	**−0.756**	**0.785**	**<0.001**
	LW	0.449	0.201	0.11	−0.257	−0.066	0.377	-	-	-
	NL	-	-	-	-	-	-	**−0.602**	**0.785**	**<0.001**
**Flatness**	LL	**−0.566**	**0.321**	**0.037**	-	-	-	**0.566**	**0.427**	**0.029**
	LW	-	-	-	0.289	0.083	0.318	-	-	-
	NL	-	-	-	-	-	-	0.479	0.427	0.056

The regression analysis was made only with the bromeliad variables retained as the best model based on the Akaike Information Criterion. The variations in the values of spider body size and flatness were partitioned into ecological (EC), niche conservatism (NCC) and phylogenetic components (PC; see text for details). The R^2^_adj_ value presents the explained variation of the best model for each component regressed against bromeliad morphological variables. Thus, the repeated R^2^_adj_ value does not represent the explained variation of each variable.

Combined with the habitat scale analysis, these results suggest that spider flatness is phylogenetically clustered at the habitat scale, whereas it is phylogenetically overdispersed at the microhabitat scale, although phylogenetic signal is present in both scales. The scale dependency of the relationship between phylogenetic relatedness and phenotypic resemblance shows that niche occupancy affects spider morphology in different ways ranging from phylogenetically clustered to overdispersed. In addition, niche occupancy seems to be very scattered in the phylogeny of spiders suggesting that habitat preference evolved several times (Fig. 1).

## Discussion

We have demonstrated that both habitat and microhabitat affect the phylogenetic and phenotypic patterns in plant-living spiders. Bromeliad-living spiders are larger and flatter than spiders foraging in the surrounding dicots. The observed morphological variation of this spider community suggests that at the habitat scale closely related spiders are selectively colonizing plants with similar architecture. However, at the microhabitat scale both selective colonization and adaptive evolution are driving changes in spider body shape. The novel contribution of our results is that even on a scale of few centimeters, plant morphological variables are sorting for spiders' body size and shape. This sorting is the outcome of the scale-dependency of the interaction between phylogenetic and ecological niches of spiders.

### Habitat Scale: Phylogenetic Signals and the Conservatism of Body Size among Guilds

At the habitat scale, we found that spiders occupying architecturally similar plant species are phylogenetically clustered. Within each habitat type, closely related spiders are more similar in body size and flatness than between habitats. Bromeliads can sort for large-bodied closely related spiders because, in general, large organisms have higher resource requirements, which constrain them from occupying neighbouring habitats with lower resource inputs. It has been demonstrated that the retention of rainwater in bromeliad phytotelmata favors the accumulation of aquatic and terrestrial invertebrates in comparison to surrounding plants that do not accumulate rainwater [7]. Thus, it is reasonable to infer that bromeliads can provide greater resources for spiders, as these animals can eat both aquatic and terrestrial prey [21]. Although spiders were larger on bromeliads, we do not have evidence that they have evolved larger while living on bromeliads, which suggests that bromeliad-spiders have selectively colonized these plants. The positive relationship between habitat quality (e.g. habitat size/complexity, prey density) and individual size was previously demonstrated for vertebrates [22] and spiders [23]. For instance, Smith et al. [24] reported that the evolution of giant terrestrial mammals was apparently influenced by ecological niches and land area in response to energy acquisition.

In addition, it is possible that the ability to dive into the water accumulated in bromeliad's phytotelmata influence spider body size. Previous works have demonstrated that large animals dive deeper, which can improve foraging efficiency by providing access to large prey and aid in avoiding predators [25], [26]; this mechanism could favor large bromeliad-living spiders, which frequently dive to capture aquatic prey and flee from predators. In fact, only large bromeliad spiders (e.g. *Corinna* sp., *Nothroctenus fuxico*, *Pachistopelma* sp., *Psecas* sp.) dive into bromeliad water, whereas young, small spiders do not (G. Q. Romero and T. Gonçalves-Souza, pers. obs.).

Bromeliads can also sort for flatter spiders, because flat bodies can enable organisms to inhabit the tight spaces between bromeliad leaf axils (even when diving), in foraging and predation avoidance. In the evolutionary history of the association between spiders and bromeliads, flatter closely related spiders could forage better among plant axils than less flat spiders. Predation may also be an important mechanism dictating body flatness. For instance, Sillett et al. [27] reported that *Pseudocolaptes lawrencii* (Furnariidae), a bird specialized in foraging in bromeliads, avoids eating isopods because they are dorsoventrally flattened, which make them difficult to catch. A flattened body has also been found in other vertebrates and invertebrates that forage in habitats with narrow spaces, such as bromeliads, caves, rocks [6], [28]–[30]. If the success of foraging in bromeliads is related to the ability to use all available leaf surfaces, spiders that are able to forage in small spaces between leaves (i.e. flatter spiders) could have advantages in terms of foraging and predator avoidance. These results are in accordance with the maneuverability hypothesis [8], which predicts a decrease in some morphological characteristics when such decreasing enhances species' performance (e.g. sexual display, foraging) particularly for species that inhabits restrictive habitats, such as bromeliads, rock crevices and dense habitats [30]. These results suggest that bromeliad architecture sort out larger and flatter closely related spiders by means of energy supply, foraging efficiency and the ability to avoid natural enemies.

Phenotypic variation among spider guilds was explained by both phylogeny and niche conservatism. This result indicates that closely related spiders have similar body sizes and belong to the same guild (Fig. 1). Within each guild, spiders share the ability to weave webs or lack thereof, which has a drastic influence on their locomotion, foraging behavior and habitat selection. In addition, spiders' foraging behavior is related to body position (i.e. upside-down walking and standing), which could generate different patterns of body size and shape between web-building and hunting spiders [31]. As a result, the phenotypic similarity in spider body sizes should be higher within than among guilds, and this resemblance most likely accounts for the strong phylogenetic signals.

### Microhabitat Scale: Phylogenetic Signals in Body Size, and the Shared Influence of Phylogeny and Ecology on Spider Flatness

At the microhabitat scale, morphological traits of spiders were related to both phylogenetic and ecological components. Bromeliad leaf length and the number of leaves had a negative correlation with the phylogenetic component of spider body size and explained 78% of its variation. This result indicates that large bromeliads select for small-bodied closely related spiders. Bromeliad leaf length was positively correlated with the phylogenetic component and negatively with the ecological component of body flatness. These results suggest that distinct evolutionary and ecological processes drive the variation in spider flatness. On the one hand, closely related spiders with flatter bodies could selectively colonize bromeliads. On the other hand, bromeliad architecture favoring the expected deviation of those ancestral values, which means that body flatness of spiders is intensified after subsequent specialization to bromeliads.

The morphological variation had phylogenetic signals also within bromeliad-living spiders. For example, closely related round (less flattened) spiders were sorted out in bromeliads with greater leaf length and so this variation could be inherited throughout the lineages. In contrast, the ecological component exhibited an inverse correlation with leaf length (Table 3), suggesting that contemporary factors are also influencing the variation in body size independently of historical factors, by independent selective pressures driving phenotypic variation in each species, as discussed above. Taken together, these results suggest that at the microhabitat scale both selective colonization of plants with specific architectures and adaptive evolution are driving the variation in spider body shape.

The occurrence of small-bodied spider on large bromeliads appears to be an intriguing pattern of body size distribution among plant-living spiders. For many animals, it is well established that body size is positively correlated with fecundity [32] and resource-rich habitats [33]. However, the developmental time necessary to achieve a large size generally increases the probability of predation, which in turn acts against the selection for larger size [34]. As a result of this trade-off, small-bodied species could benefit from occupying large bromeliads because they can more effectively avoid predation by finding more retreats. In fact, it has been suggested that small animals are more agile and maneuverable [8], [34]. Otherwise, where spiders occur in larger habitats/microhabitats, it is reasonable that large bromeliads should support a greater number of small spiders than large ones. Thus, we suggest that predation and the ability to support more small spiders are not mutually exclusive factors; it is possible that they work together constraining the increase in body size of spiders within bromeliads.

The negative correlation between the ecological component (guild) and bromeliad leaf length suggests that bromeliad spiders' flatness is most likely an adaptive response to the tight arrangement of bromeliad leaves. Patterns of spider body size/shape evolution (or conservatism) could arise from both habitat selection and competitive ability, depending on either the ecological relevance of species' traits or the scale considered (e.g. regional or local, habitat or microhabitat).

Thus, differences in habitat and microhabitat characteristics have contrasting evolutionary pathways in spider-plant association. On the one hand, differences in habitat scale favor the occurrence of closely related spiders of similar size (i.e. phylogenetic clustering), most likely because these spiders share similar ecological requirements, such as energetic requirements. In contrast, to better explore resources in tightly arranged microhabitats while escaping from predators, it appears that microhabitat constraints favor the evolution of flatter spiders on bromeliads.

## Conclusion

Our results showed that spider flatness is phylogenetically clustered at the habitat scale, whereas it is phylogenetically overdispersed at the microhabitat scale, although phylogenic signal is present in both scales. We found evidence that species' evolutionary history is a result of several processes (selective colonization, adaptive evolution) working at different scales. Maybe more importantly, we have shown that the relative importance of these processes change with scale, which adds voice to Swenson et al. [35] that argue that studies considering the scale dependency of ecological and evolutionary processes could found hidden patterns of (phylogenetic) community structure. Besides considering local and regional influence as suggested by Swenson et al. [35], our study emphasized that by decoupling “local” scale in finer scales such as habitat and microhabitat, one can found “hidden” ecological and evolutionary patterns that were not evident in larger scales.

## Supporting Information

File S1 Supporting tables.
(DOCX)
