# Correction: Disentangling the Phylogenetic and Ecological Components of Spider Phenotypic Variation

**DOI:** 10.1371/journal.pone.0097978

**Published:** 2014-05-12

**Authors:** The PLOS ONE Staff

The legends for Figure 1 and Figure 2 are reversed. The authors have also chosen to provide higher-quality images for Figure 2 and Figure 3. Please view the correct images and legends for Figure 1, Figure 2, and Figure 3 here.

**Figure 1:**
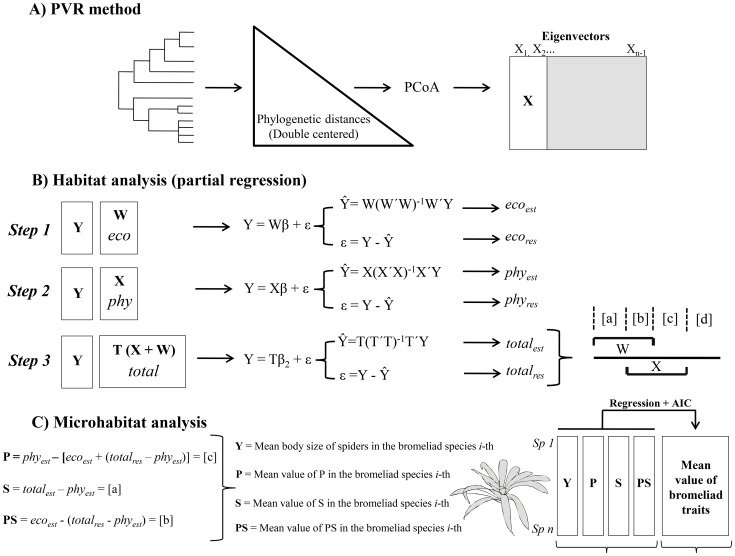
Schematic representation of the habitat and microhabitat analyses used to decompose the total variation in spider body size and flatness into phylogenetic, ecological and niche conservatism components. Phylogenetic eigenvector regression (PVR) is represented by a back-transformation of the phylogeny with a double-centralization of the resulting matrix and is followed by a principal coordinates analysis (PCoA); the matrix X represents the eigenvectors that are significantly correlated with species’ body size (Fig. 2A). Figure 2B shows the partial regressions used to calculate components a, b, c and d; first, we calculated the estimated and residual values (*eco_est_* and *eco_res_*) for a regression between body size and the ecological data; then, we regressed body size and the phylogenetic data and saved the estimated and residual values (*phy_est_* and *phy_res_*); finally, we computed the regression between body size and both the ecological and the phylogenetic data to obtain the percentages of the variance explained (R2 of the regression method) by the ecological component [a], the niche conservatism b, the phylogenetic component [c] (phylogeny) and the unexplained variation d (unexplained variation), following the procedure proposed by Desdevises et al. [15]. Figure 2C illustrates the procedure used to obtain the mean value of spider body size (or flatness) and the average value of components [a], [b] and [c] obtained (see Figure 2B) for each bromeliad species. We then constructed a linear regression between each value (Y, [a], [b] and [c]) and the mean value of bromeliad morphological variables (leaf length, leaf width and number of leaves) after the selection of best models with the Akaike Information Criterion (AIC).

**Figure 2:**
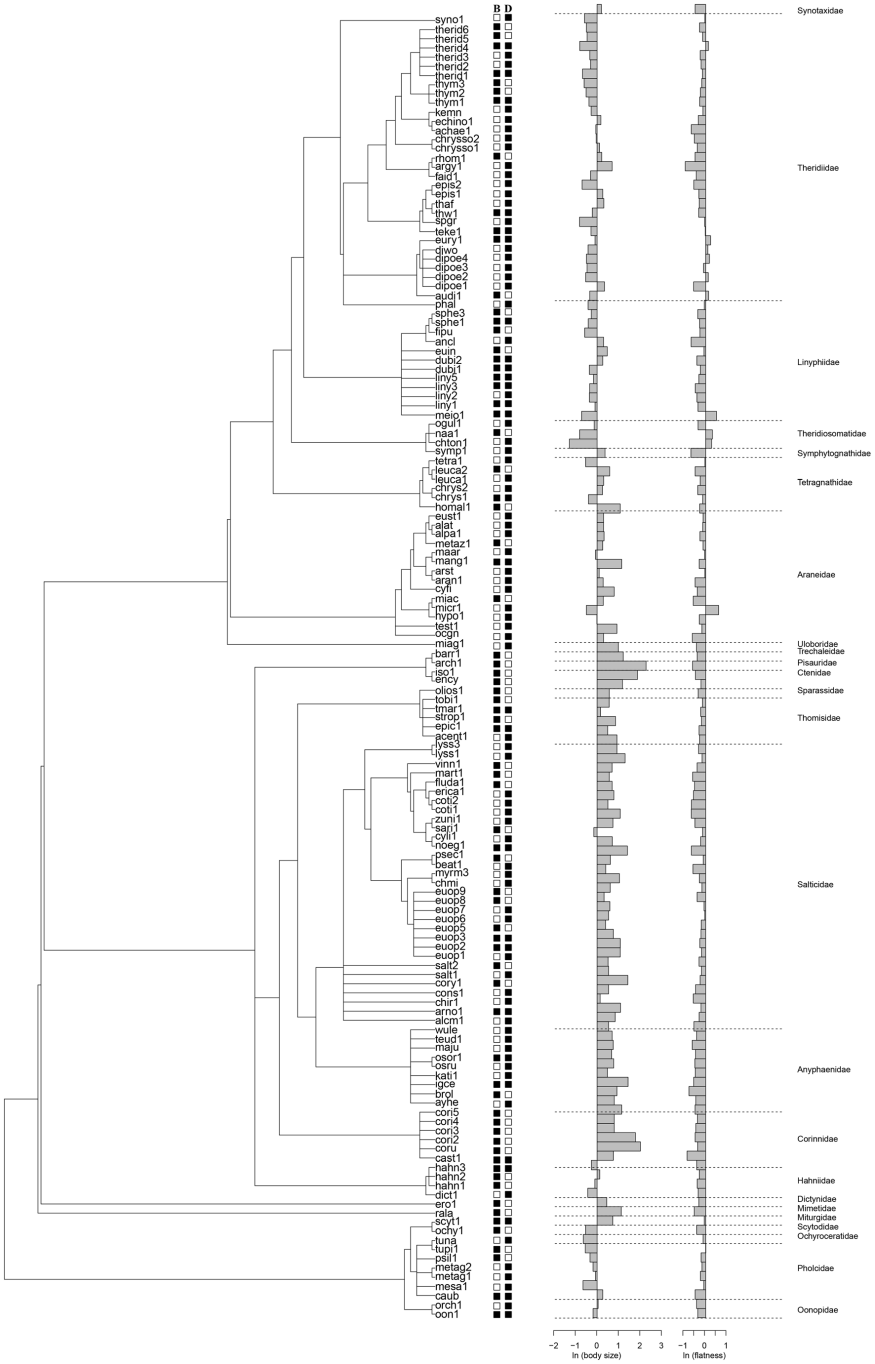
Consensus topology describing the phylogenetic relationships among spider species. Presence (black squares) and absence (white squares) of spider species in bromeliads (B) and/or dicot plants (D) (middle panel). The right panel shows the body size and flatness of species (grey bars) in logarithmic scale.

**Figure 3:**
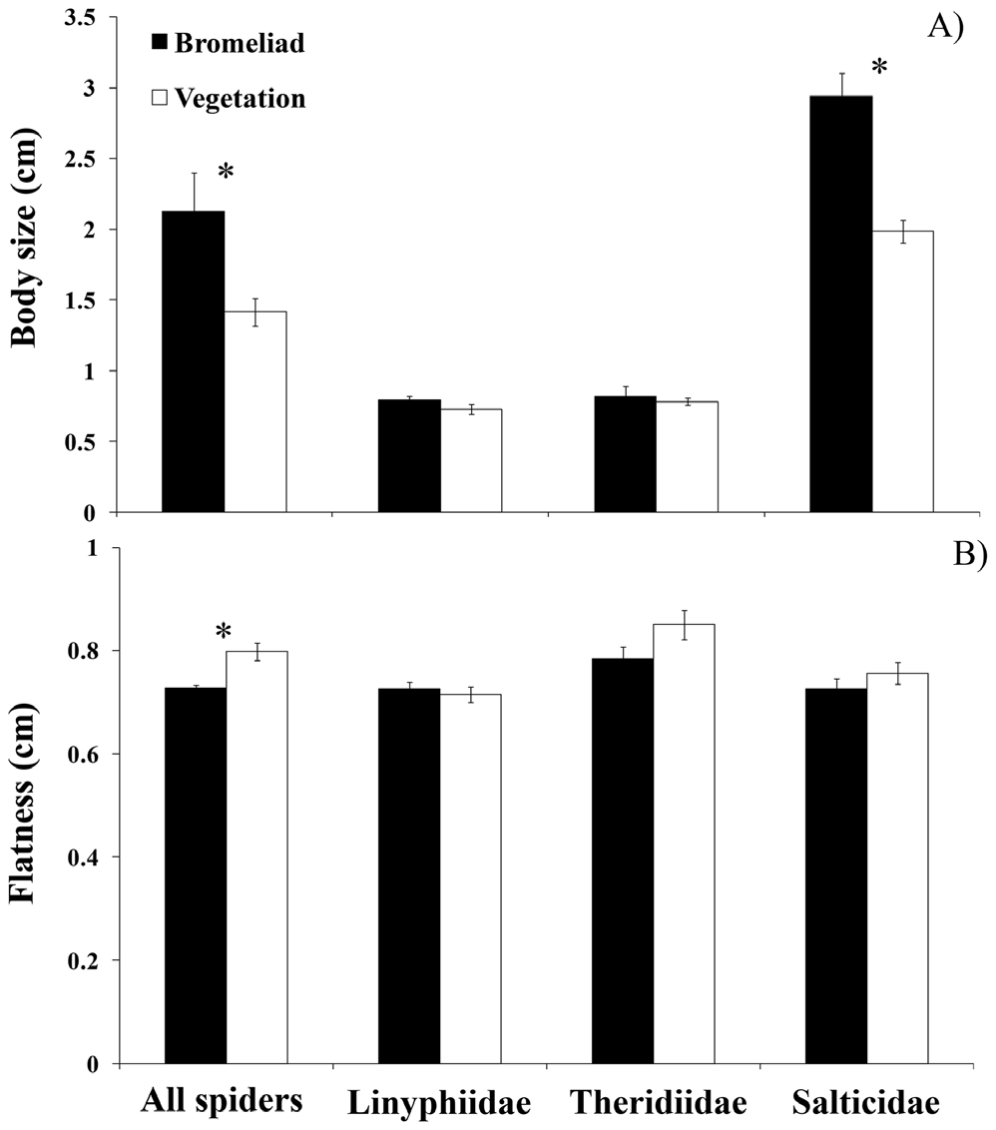
Average spider body size (A) and flatness (B) between bromeliads and surrounding dicots for all spiders, Linyphiidae, Theridiidae (both families of web-spiders) and Salticidae (hunting spiders). Error bars denote ± SE and asterisks indicate significant difference (P  =  0.05).
