# Supplementary material for: Disentangling the Phylogenetic and Ecological Components of Spider Phenotypic Variation
Source: PLoS One. 2014 Feb 19;9(2):e89314. doi: 10.1371/journal.pone.0089314 (PMC3942061; doi:10.1371/journal.pone.0089314)
Supplement: File S1 — Supporting tables. (DOCX) [file pone.0089314.s001.docx]

**SUPPORTING INFORMATION**

**Disentangling the phylogenetic and ecological components of spider phenotypic variation**

Thiago Gonçalves-Souza, José Alexandre F. Diniz-Filho & Gustavo Q. Romero

**Phylogeny construction and spider guilds**

We compiled data from morphological and molecular phylogenies of spiders to create one unique tree, which was used to estimate the phylogenetic (patristic) distance among species. We used data from a general phylogeny (Coddington and Levi 1991) and other phylogenies made at the family or superfamily levels: Agnarsson (2004; family Theridiidae), Álvarez-Padilla et al. (2009: Tetragnathidae), Griswold et al. (1998; Orbicularie: Deinopoidea, Araneoidea), Hedin and Maddison (2001; Salticidae), Hormiga (1994; Linyphiidae), and Maddison and Hedin (2003; Salticidae). The composite tree was based on the 145 spider species observed in this study occurring in either bromeliad or dicot (herbaceous and shrubby plants) habitats. We based our tree on the general phylogeny of Coddington and Levi (1991) and attributed the longest node at the family level to ensure that genera and species were more similar within families than between families. Within each family with a defined phylogeny (references above), we organised the genera and species based on their proposed kinship. For example, Maddison and Hedin (2003) proposed the genera *Sarinda*, *Zuniga* and *Agelista* as sister groups. Thus, as we collected one species of *Sarinda* and one of *Zuniga* but no *Agelista* species, we defined *Sarinda* sp. and *Zuniga* sp. as a monophyletic group in our composite tree. We considered as monophyletic and polytomous (Vamosi and Vamosi 2007) those families (e.g., Corinnidae, Hahniidae) or genera (e.g., Corinna, Theridion) without phylogenetic information. As the species we collected were not dimorphic (Table S2), the body sizes of males and females were averaged for each species. We used the program PDTREE to draw the phylogenetic tree and PDDIST to calculate phylogenetic distances (Garland and Ives 2000).

| **Table S1.** Spider species as assigned to families and guilds | | |
| --- | --- | --- |
| **Species** | **Family** | **Guild** |
| *Oonops* sp.1 | Oonopidae | hunting spiders |
| *Orchestina* sp.1 | Oonopidae | hunting spiders |
| *Carapoia ubatuba* Huber, 2005 | Pholcidae | web-building spiders |
| *Mesabolivar* sp.1 | Pholcidae | web-building spiders |
| *Metagonia* sp.1 | Pholcidae | web-building spiders |
| *Metagonia* sp.2 | Pholcidae | web-building spiders |
| *Psilochorus* sp.1 | Pholcidae | web-building spiders |
| *Tupigea* sp.1 | Pholcidae | web-building spiders |
| *Tupigea nadleri* Huber, 2000 | Pholcidae | web-building spiders |
| *Ochyrocera* sp.1 | Ochyroceratidae | web-building spiders |
| *Scytodes* sp.1 | Scytodidae | hunting spiders |
| *Radulphius laticeps* | Miturgidae | hunting spiders |
| *Ero* sp.1 | Miturgidae | hunting spiders |
| *Dictyna* sp.1 | Dictynidae | web-building spiders |
| Hahniidae sp.1 | Hahniidae | hunting spiders |
| Hahniidae sp.2 | Hahniidae | hunting spiders |
| Hahniidae sp.3 | Hahniidae | hunting spiders |
| *Castianeira* sp.1 | Corinnidae | hunting spiders |
| *Corinna rubripes* | Corinnidae | hunting spiders |
| *Corinna* sp.2 | Corinnidae | hunting spiders |
| *Corinna* sp.3 | Corinnidae | hunting spiders |
| *Corinna* sp.4 | Corinnidae | hunting spiders |
| *Corinna* sp.5 | Corinnidae | hunting spiders |
| *Aysha* gr. *helvola* | Anyphaenidae | hunting spiders |
| *Bromelina oliola* Brescovit, 1993 | Anyphaenidae | hunting spiders |
| *Iguarima censoria* Keyserling, 1891 | Anyphaenidae | hunting spiders |
| *Katissa* sp.1 | Anyphaenidae | hunting spiders |
| *Osoriella rubella* Keyserling, 1891 | Anyphaenidae | hunting spiders |
| *Osoriella* sp.1 | Anyphaenidae | hunting spiders |
| *Macrophyes jundiai* Brescovit, 1993 | Anyphaenidae | hunting spiders |
| *Teudis* sp.1 | Anyphaenidae | hunting spiders |
| *Wulfilopsis leopoldina* Brescovit, 1997 | Anyphaenidae | hunting spiders |
| *Alcmena* sp.1 | Salticidae | hunting spiders |
| *Arnoliseus* sp.1 | Salticidae | hunting spiders |
| *Chirothecia* cf. sp.1 | Salticidae | hunting spiders |
| *Consingis* sp.1 | Salticidae | hunting spiders |
| *Coryphasia* sp.1 | Salticidae | hunting spiders |
| Salticidae sp.1 | Salticidae | hunting spiders |
| Salticidae sp.2 | Salticidae | hunting spiders |
| Euophryinae sp.1 | Salticidae | hunting spiders |
| Euophryinae sp.2 | Salticidae | hunting spiders |
| Euophryinae sp.3 | Salticidae | hunting spiders |
| Euophryinae sp.5 | Salticidae | hunting spiders |
| Euophryinae sp.6 | Salticidae | hunting spiders |
| Euophryinae sp.7 | Salticidae | hunting spiders |
| Euophryinae sp.8 | Salticidae | hunting spiders |
| Euophryinae sp.9 | Salticidae | hunting spiders |
| *Chira micans* Simon, 1902 | Salticidae | hunting spiders |
| *Myrmarachne* sp.3 | Salticidae | hunting spiders |
| *Beata* sp.1 | Salticidae | hunting spiders |
| *Psecas* sp.1 | Salticidae | hunting spiders |
| *Noegus* sp.1 | Salticidae | hunting spiders |
| *Cylistella* sp.1 | Salticidae | hunting spiders |
| *Sarinda* sp.1 | Salticidae | hunting spiders |
| *Zuniga* sp.1 | Salticidae | hunting spiders |
| *Cotinusa* sp.1 | Salticidae | hunting spiders |
| *Cotinusa* sp.2 | Salticidae | hunting spiders |
| *Erica* sp.1 | Salticidae | hunting spiders |
| *Fluda* sp.1 | Salticidae | hunting spiders |
| *Martella* sp.1 | Salticidae | hunting spiders |
| *Vinnius* sp.1 | Salticidae | hunting spiders |
| *Lyssomanes* sp.1 | Salticidae | hunting spiders |
| *Lyssomanes* sp.3 | Salticidae | hunting spiders |
| *Acentroscelus* sp.1 | Thomisidae | hunting spiders |
| *Epicadus* sp.1 | Thomisidae | hunting spiders |
| *Strophius* sp.1 | Thomisidae | hunting spiders |
| *Tmarus* sp.1 | Thomisidae | hunting spiders |
| *Tobias* sp.1 | Thomisidae | hunting spiders |
| *Olios* sp.1 | Sparassidae | hunting spiders |
| *Enoploctenus cyclothorax* Bertkau, 1880 | Ctenidae | hunting spiders |
| *Isoctenus* sp.1 | Ctenidae | hunting spiders |
| *Architis* sp.1 | Pisauridae | hunting spiders |
| *Barrisca* sp.1 | Trechaleidae | web-building spiders |
| *Miagrammopes* sp.1 | Uloboridae | web-building spiders |
| *Ocrepeira gnomo* Mello-Leitão, 1943 | Araneidae | web-building spiders |
| *Testudinaria* sp.1 | Araneidae | web-building spiders |
| *Hypognatha* sp.1 | Araneidae | web-building spiders |
| *Micrathena* sp.1 | Araneidae | web-building spiders |
| *Micrathena acuta* Walckenaer, 1842 | Araneidae | web-building spiders |
| *Cyclosa fililineata* Hingston, 1932 | Araneidae | web-building spiders |
| *Araneus* sp.1 | Araneidae | web-building spiders |
| *Araneus stabilis* Keyserling, 1892 | Araneidae | web-building spiders |
| *Mangora* sp.1 | Araneidae | web-building spiders |
| *Mangora aripeba* | Araneidae | web-building spiders |
| *Metazygia* sp.1 | Araneidae | web-building spiders |
| *Alpaida* sp.1 | Araneidae | web-building spiders |
| *Alpaida atomaria* Simon, 1895 | Araneidae | web-building spiders |
| *Eustala* sp.1 | Araneidae | web-building spiders |
| *Homalometa* sp.1 | Araneidae | web-building spiders |
| *Chrysometa* sp.1 | Tetragnathidae | web-building spiders |
| *Chrysometa* sp.2 | Tetragnathidae | web-building spiders |
| *Leucauge* sp.1 | Tetragnathidae | web-building spiders |
| *Leucauge* sp.2 | Tetragnathidae | web-building spiders |
| *Tetragnatha* sp.1 | Tetragnathidae | web-building spiders |
| *Symphytognatha* sp.1 | Symphytognathidae | web-building spiders |
| *Chthonos* sp.1 | Theridiosomatidae | web-building spiders |
| *Naatlo* sp.1 | Theridiosomatidae | web-building spiders |
| *Ogulnius* sp.1 | Theridiosomatidae | web-building spiders |
| *Meioneta* sp.1 | Linyphiidae | web-building spiders |
| Linyphiidae sp.1 | Linyphiidae | web-building spiders |
| Linyphiidae sp.2 | Linyphiidae | web-building spiders |
| Linyphiidae sp.3 | Linyphiidae | web-building spiders |
| Linyphiidae sp.5 | Linyphiidae | web-building spiders |
| *Dubiaranea* sp.1 | Linyphiidae | web-building spiders |
| *Dubiaranea* sp.2 | Linyphiidae | web-building spiders |
| *Eurymorion insigne* Millidge, 1991 | Linyphiidae | web-building spiders |
| *Anodoration claviferum* Millidge, 1991 | Linyphiidae | web-building spiders |
| *Fissiscapus pusillus* Millidge, 1991 | Linyphiidae | web-building spiders |
| *Sphecozone* sp.1 | Linyphiidae | web-building spiders |
| *Sphezocone* sp.3 | Linyphiidae | web-building spiders |
| *Phycosoma altum* Keyserling, 1886 | Theridiidae | web-building spiders |
| *Audifia* sp.1 | Theridiidae | web-building spiders |
| *Dipoena* sp.1 | Theridiidae | web-building spiders |
| *Dipoena* sp.2 | Theridiidae | web-building spiders |
| *Dipoena* sp.3 | Theridiidae | web-building spiders |
| *Dipoena* sp.4 | Theridiidae | web-building spiders |
| *Dipoena woytkowskii* Levi, 1963 | Theridiidae | web-building spiders |
| *Euryopis* sp.1 | Theridiidae | web-building spiders |
| *Tekellina* sp.1 | Theridiidae | web-building spiders |
| *Spintharus gracilis* Keyserling, 1886 | Theridiidae | web-building spiders |
| *Thwaithesia* sp.1 | Theridiidae | web-building spiders |
| *Thwaitesia affinis* O.P. Cambridge, 1882 | Theridiidae | web-building spiders |
| *Episinus* sp.1 | Theridiidae | web-building spiders |
| *Episinus* sp.2 | Theridiidae | web-building spiders |
| *Faiditus* sp.1 | Theridiidae | web-building spiders |
| *Argyrodes* sp.1 | Theridiidae | web-building spiders |
| *Rhomphae* sp.1 | Theridiidae | web-building spiders |
| *Chrysso* sp.1 | Theridiidae | web-building spiders |
| *Chrysso* sp.2 | Theridiidae | web-building spiders |
| *Achaearanea* sp.1 | Theridiidae | web-building spiders |
| *Echinotheridion* sp.1 | Theridiidae | web-building spiders |
| *Keijia mneon* Bösenberg & Strand, 1906 | Theridiidae | web-building spiders |
| *Thymoites* sp.1 | Theridiidae | web-building spiders |
| *Thymoites* sp.2 | Theridiidae | web-building spiders |
| *Thymoites* sp.3 | Theridiidae | web-building spiders |
| *Theridion* sp.1 | Theridiidae | web-building spiders |
| *Theridion* sp.2 | Theridiidae | web-building spiders |
| *Theridion* sp.3 | Theridiidae | web-building spiders |
| *Theridion* sp.4 | Theridiidae | web-building spiders |
| *Theridion* sp.5 | Theridiidae | web-building spiders |
| *Theridion* sp.6 | Theridiidae | web-building spiders |
| *Synotaxus* sp.1 | Synotaxidae | web-building spiders |

| **Table S2**. Ratio between female and male prosoma size of each species with at least one individual of both sexes. Species with values of female/male ratio less than 0.5 and higher than 2 were considered dimorphic^*1^. | |
| --- | --- |
| **Spider species** | **Prosoma size ratio** |
| *Anodoration claviferum* Millidge, 1991 | 0.899 |
| *Arnoliseus* sp.1 | 1.770 |
| *Barrisca* sp.1 | 1.016 |
| *Beata* sp.1 | 0.757 |
| *Castianeira* sp.1^*2^ | 1.117 |
| *Castianeira* sp.1 | 1.180 |
| *Chrysometa* sp.2 | 0.879 |
| *Chrysso* sp.1 | 0.908 |
| *Chrysso* sp.2 | 0.783 |
| *Chthonus* sp.1 | 0.946 |
| *Coryphasia* sp.1 | 0.895 |
| *Cotinusa* sp.1 | 0.950 |
| *Cotinusa* sp.2 | 1.359 |
| *Cyclosa fililineata* Hingston, 1932 | 0.945 |
| *Cylistella* sp.1 | 0.983 |
| *Dipoena* sp.1 | 1.048 |
| *Dipoena* sp.3 | 1.052 |
| *Epicadus* sp.1 | 1.500 |
| Euophryinae sp.3 | 0.726 |
| *Fissiscapus pusillus* Millidge, 1991 | 1.141 |
| Hahniidae sp.1 | 0.868 |
| Hahniidae sp.2 | 0.910 |
| Hahniidae sp.3 | 1.093 |
| Linyphiidae sp.1 | 0.831 |
| Linyphiidae sp.1 | 1.049 |
| Linyphiidae sp.3 | 0.793 |
| *Mesabolivar* sp.1 | 0.917 |
| *Miagrammopes* sp.1 | 1.231 |
| *Noegus* sp.1 | 0.928 |
| *Oonops* sp.1 | 0.820 |
| *Phycosoma altum* Keyserling, 1886 | 0.698 |
| *Psilochorus* sp.1 | 0.970 |
| *Rhomphaea* sp.1 | 1.309 |
| *Scytodes* sp.1 | 0.843 |
| *Sphecozone* sp.1 | 0.905 |
| *Spintharus gracilis* Keyserling, 1886 | 0.945 |
| *Testudinaria* sp.1 | 1.386 |
| Tetragnatha sp.1 | 0.943 |
| *Theridion* sp.1 | 1.284 |
| *Theridion* sp.2 | 1.001 |
| *Theridion* sp.4 | 1.109 |
| *Thymoites* sp.1 | 0.868 |
| *Tmarus* sp.1 | 1.120 |
| *Vinnius* sp.1 | 1.091 |

* 1 – These cutoff values to decide which species are considered dimorphic was based on Hormiga et al. 2000.

* 2 – Some species have two values of dimorphism because their occurred at bromeliad and dicot.

**References**

Agnarsson, I. (2004) Morphological phylogeny of cobweb spiders and their relatives (Araneae, Araneoidea, Theridiidae). Zoological Journal of the Linnean Society 141: 447–626.

Álvarez-Padilla F., Dimitrov, D., Giribet, G. And Hormiga, G. (2009). Phylogenetic relationships of the spider family Tetragnathidae (Araneae, Araneoidea) based on morphological and DNA sequence data. Cladistics 25: 109-146.

Coddington J.A. and Levi H.W. (1991) Systematics and evolution of spiders (Araneae). Annual Review of Ecology and Systematics 22:565-92.

Garland, T.Jr. & Ives A.R. (2000) Using the past to predict the present: Confidence intervals for regression equations in phylogenetic comparative methods. American Naturalist 155: 346–364.

Griswold, C.E., Coddington, J.A., Hormiga, G. and Scharff, N. (1998). Phylogeny of the orb-web building spiders (Araneae, Orbiculariae: Deinopoidea, Araneoidea). Zoological Journal of Linnean Society 123: 1-99.

Hedin, M.C. and Maddison, W.P. (2001). A combined molecular approach to phylogeny of the jumping spider subfamily Dendryphantinae (Araneae: Salticidae). Molecular Phylogenetics and Evolution 18: 386-403.

Hormiga, G. (1994). Cladistics and the comparative morphology of linyphiidae spiders and their relatives (Araneae, Araneoidea, Linyphiidae). Zoological Journal of the Linnean Sociey 111: 1-71.

Hormiga G, Scharff N & Coddington JA (2000) The phylogenetic basis of sexual size dimorphism in orb-weaving spiders (Araneae, Orbiculariae). Systematic Biology **49**: 435-462.

Maddison, W.P. and Hedin, M.C. (2003). Jumping spider phylogeny (Araneae: Salticidae). Invertebrate Systematics 17: 529-549.

Vamosi, J.C. and Vamosi, S.M. (2007). Body size, rarity, and phylogenetic community structure: insights from diving beetle assemblages of Alberta. Diversity and Distributions 13: 1-10.
